# The current landscape of nucleic acid tests for filovirus detection

**DOI:** 10.1016/j.jcv.2018.03.005

**Published:** 2018-06

**Authors:** David J. Clark, John Tyson, Andrew D. Sails, Sanjeev Krishna, Henry M. Staines

**Affiliations:** a Centre for Diagnostics & Antimicrobial Resistance, Institute for Infection & Immunity, St. George’s University of London, Cranmer Terrace, London SW17 0RE, UK; b Institute for Infection & Immunity, St. George’s University of London, Cranmer Terrace, London SW17 0RE, UK; c QuantuMDx, Lugano Building, 57 Melbourne Street, Newcastle-upon-Tyne, NE1 2JQ, UK; d St. George’s University Hospitals NHS Foundation Trust, Blackshaw Road, Tooting, London SW17 0QT, UK

**Keywords:** Filoviridae, Ebola, Marburg, Diagnostics, Point-of-care, NAT

## Abstract

Nucleic acid testing (NAT) for pathogenic filoviruses plays a key role in surveillance and to control the spread of infection. As they share clinical features with other pathogens, the initial spread of these viruses can be misdiagnosed. Tests that can identify a pathogen in the initial stages of infection are essential to control outbreaks. Since the Ebola virus disease (EVD) outbreak in 2014–2016 several tests have been developed that are faster than previous tests and more suited for field use. Furthermore, the ability to test for a range of pathogens simultaneously has been expanded to improve clinical pathway management of febrile syndromes. This review provides an overview of these novel diagnostic tests.

## Highlights

• Filoviruses can cause severe hemorrhagic fever in humans and non-human primates.
• There is an urgent need for rapid diagnosis of filoviruses during outbreaks.
• Filovirus diagnostics have advanced since the 2014–2016 Ebolavirus outbreak.
• NATs are the gold standard for filovirus detection.
• NAT-based diagnostic speed, portability and multiplexing have all improved.

## 1 Introduction

**Fig. 1:**
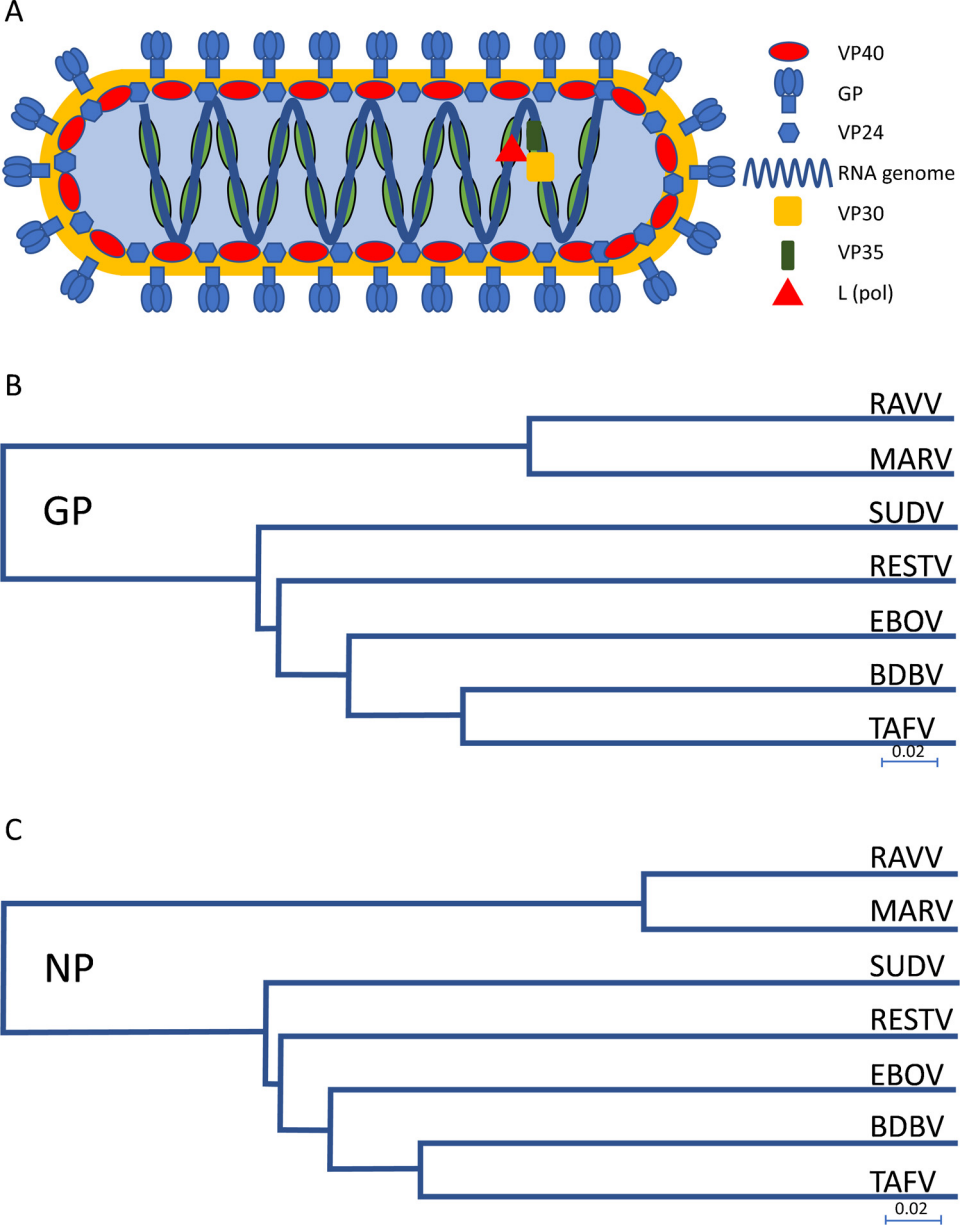
Structure and filovirus divergence. A. Schematic of a filovirus particle. Nucleotide sequence phylogenetic trees, prepared with NCBI genomics workbench using nearest neighbour joining, showing the main targets GP (B) and NP (C) for filovirus NATs. The divergence in sequence requires specific primers for each species/subspecies. Scale bar denotes nucleotide substitutions per site.

The Ebolaviruses are a group of closely related viruses in the *filoviridae* family. Filoviruses have negative-sense RNA genomes protected by the nucleocapsid protein (Fig. 1A). There are five distinct Ebolavirus species; Bundibugyo (BDBV), Reston (RESTV), Sudan (SUDV), Taï forest (TAFV, formerly Cote d’Ivoire Ebolavirus) and Zaire (EBOV). All five members can cause infections in humans albeit with a wide spectrum of disease severity. The Zaire and Sudan species cause Ebolavirus Disease (EVD) with a fatality rate ranging from 40 to 90% [1]. These viruses, along with other more distantly related filoviruses, Marburg virus (MARV) and Ravn virus (RAVV), are biosafety level 4 agents associated with high fatality rates and an absence of effective treatments [2].

The first documented Ebolavirus, and prototypic virus for the group, EBOV, was isolated in an outbreak of a haemorrhagic fever in Africa within the democratic Republic of Congo (formerly Zaire) in 1976 [3], nine years after MARV had been discovered in a laboratory-centred outbreak in Marburg, Germany. Both TAFV and RESTV are somewhat unusual within the Ebolavirus group as firstly; they do not appear to cause severe pathology in humans, (in non-human primates, infections are highly pathogenic) and secondly the sources were outside the central African continental region associated with filovirus outbreaks.

Filoviruses are considered to be zoonotic; there is evidence that bats are likely to be an animal reservoir for a number of viruses. Bats can harbour Ebolaviruses and Marburg virus, which replicate without causing symptoms of EVD [4]; filovirus RNA has been isolated from 3 species of bat [5,6] and more species have been shown to have antibodies against Ebolavirus [[7], [8], [9], [10], [11]]. Evidence suggests that contact between humans and fruit bats are the cause of at least one outbreak [12]. Furthermore, experiments have shown that pigs infected with EBOV can transmit virus to non-human primates kept in the same room but with no physical contact [13].

In March 2014 an outbreak of the EBOV began in western Africa. This was the largest outbreak recorded and spanned several countries in the region. Initially there were relatively few cases but they rapidly increased as transmission started to occur in densely populated areas. In 2014 the World Health Organisation (WHO) declared the epidemic as a Public Health Emergency of International Concern (PHEIC) [14]. The PHEIC was declared over, in March 2016 after the three countries, that were the main focus of the international response, completed 42 days with no newly reported cases and an additional 90 days of enhanced surveillance [15]. During the 2-year outbreak, there were more than 28,600 suspected, possible or confirmed cases of which 11,310 were fatal (∼40% case fatality rate) [16]. This epidemic highlighted the need for rapid diagnostics and epidemiology for disease tracking and containment. The unprecedented scale of the EVD outbreak spurred research into the filovirus field with the swift deployment of experimental vaccines, for phase I/II [[17], [18], [19], [20], [21], [22], [23], [24], [25], [26], [27]] and III trials [28], and development of diagnostics suited for low resource and in-field environments.

Future development of diagnostics focusing on rapid, sensitive and specific assays will be especially helpful in triage, as the symptoms of early EVD overlap with several other infections found in equatorial regions (e.g. malaria). Early isolation of EBOV infected individuals decreases the chance of perpetuating infections by breaking transmission chains. In this review, current and proposed methods and techniques for Ebolavirus diagnosis using nucleic acids (see Table 1 for advantages and disadvantages) will be described. Other technologies are not covered (e.g. ELISA, cell culture, EM).

**Table 1:** Advantages and disadvantages of NATs.

Advantages	Disadvantages
The ability to diagnose an infection prior to the adaptive immune response	Small window of diagnosis (viraemia first detectable between 3 and 10 days of initial infection) compared to antigen and antibody tests
A high specificity and multiplex compatibility	RNA is susceptible to rapid degradation by RNases
Greater sensitivity; most NATs amplify an initial sample	Cannot identify past infections, unlike antibody tests
Reduced operator handling (giving enhanced safety)	PCR product contamination risk due to amplification of initial sample
No requirement for category 4 level cell culture	Pathogen genetic drift could result in decreased sensitivity (If using DNA primers/capture probes)
Speed (with the exception of antigen RDTs)	
Definitive of virus presence; in the absence of a viral genome, there is no amplification	

## 2 Sample collection and storage

For most of the diagnostic methods used to determine the presence of virus (or evidence of contact with Ebolavirus and closely related filoviruses) a blood sample is required. WHO recommendations for venepuncture, in cases of suspected EBOV or MARV, state that blood should be collected into EDTA tubes with a minimum volume of 5 ml. For blood collection guidelines please refer to: http://www.who.int/csr/resources/publications/ebola/blood-collect/en/. The WHO guidelines further state that blood samples can be stored for up to 24 h at room temperature, or at 0–5° C for up to a week. For periods of longer than a week, the sample should be stored at −20 or −70° C (avoiding freeze thaw cycles) [29].

Oral swab collection has been tested in a guinea pig model of EVD [30]. The authors considered the test to be poor for samples collected *ante mortem* but excellent for *post mortem* specimens. Oral swab collection during the 2014–2016 west Africa outbreak has also been examined for *post mortem* surveillance [31]. The same study tested finger-stick sampling with pipette or swab collection, which in field point-of-care applications may be more feasible than venepuncture.

## 3 Sample preparation

As with all PCR-based assays, the purity of the input template is important for standardising tests. Due to inhibitors found in blood (reviewed in [32]) most diagnostic tests require the genome of a filovirus to be isolated from a sample; for example, to obtain a viraemia, RNA needs to be isolated from the blood plasma. One frequently used method utilises acid guanidinium thiocyanate-phenol-chloroform to separate a sample into an aqueous (containing RNA) and organic phase (containing DNA). This also has the important advantage of inactivating infectious material as proteins are denatured, although for complete inactivation of Ebolavirus, a secondary ethanol step appears to be necessary [33]. However, this method can be impracticable as it is both time consuming for an operator and uses harmful chemicals.

Commercial RNA extraction kits, many of which are based on chaotropic chemical (e.g. guanidine-isothiocyanate) extraction, use centrifugation (or vacuum) of columns containing glass fiber filters to isolate RNA, allowing batch processing. Other kits use beads, with high nucleic acid adherence, followed by magnetic separation. Most kits can concentrate nucleic acids which has the potential of improving the sensitivity of downstream assays.

Due to the sensitivity of RNA to RNases, care must be taken to minimize degradation of extracted samples prior to reverse transcription. However, there is now a move towards using all-in-one RNA isolation and reaction devices, as this minimizes the exposure of sample to RNases and of staff to potentially infectious material and reduces operator dependent variation allowing for more standardized tests.

## 4 Diagnostics

All members of the *filoviridae* have negative-sense RNA genomes and therefore require a reverse transcription step before polymerase chain reaction (PCR) can be performed. There are three main reverse transcriptase PCR techniques, as described below.

### 4.1 RT-PCR

There are two main methods of reverse transcriptase PCR; one step and two step. In one step RT-PCR, the sample and all required reagents are within one reaction chamber/tube and an initial reverse transcription step is directly followed by PCR cycling. cDNA libraries of total RNA in a sample can be prepared using random primers, or specific pathogen primers can be used; thereby only generating cDNA of a particular pathogen.

In two step RT-PCR, the reverse transcription and PCR are performed separately; the first to generate cDNA and the second for PCR, following the transfer of template cDNA to another chamber. With a cDNA library of total RNA, two step RT-PCR the resulting template can be used in multiple separate assays but is time consuming and sensitivity can be reduced due to splitting of the original sample.

In both one step and two step RT-PCR, the final products are assessed by agarose gel and is therefore more suited to qualitative diagnosis. The main advantage of this technique, compared to RT-qPCR, is the cost; equipment to perform PCR and electrophoresis are relatively inexpensive. However, it is not possible to multiplex within an assay; while there is some RNA sequence conservation within a species of filovirus there is far less between species (Fig. 1B and C). Published RT-PCR assays are presented in Table 2.

**Table 2:** RT-PCR papers; targets, primers and sensitivity for filovirus detection.

Paper	Target gene/sequence	Primers	Sensitivity*
Leroy [69]	Polymerase, L	Zaire F 5′- ATCGGAATTTTCTTTCTCATTGAAAGA-3′	100% (30/30) (95% CI 88.4–100)**
		Zaire R 5′- ATGTGGTGGATTATAATAATCACTGACATGCAT-3′	
Towner [70]	Nucleoprotein, NP	Primary	N/A
		SudZaiNP1(F), 5′-GAGACAACGGAAGCTAATGC-3′,	
		SudZaiNP1(R), 5′-AACGGAAG ATCACCATCATG-3′,	
		Nested	10^4^–10^5^/mL (10–100/reaction)
		SudZaiNP2(F), 5′-GGTCAGT TTCTATCCTTTGC-3′,	
		SudZaiNP2(R), 5′-CATGTGTCCAACTGATTG CC-3′	
Park [71]	NP	BDBV F GCAGAAATATGCTGAATCTCGTGAAC	5 fg/μL
		BDBV R ATCATCCTCGTCCTCAAGGTCAAAA	
		RESTV F CCAACAATATGCTGAGTCCAGAGAA	5 fg/μL
		RESTV R	
		CATCCTCATGATCGTCAAGATCG	
		SUDV F ACACGTGAGTTGGACAACCTT	5 fg/μL
		SUDV R GTCATCGTCGTCGTCCAAATTGAA	
		TEBOV F AATCTCGCGAGCTTGACCAT	5 fg/μL
		TEBOV R CTCGTCACCATCTTCAAGGTCAAA	
		EBOV F CGAACTTGACCATCTTGGACTTG	5 fg/μL
		EBOV R TCCTCGTCGTCCTCGTCTAGAT	
		MARV F AGGCGACATGAACATCAGGAAATT	5 fg/μL
		MARV R TCGTCCTCATTCAGCAGTGCAAAT	
		RAVV F GCGACATGAACACCAGGAAATTC	500 fg/μL
		RAVV R ATTTTCAAGAGTATCCTCGTCTTCG	
Ogawa 2011[72]	NP	MARV FiloNP-Fm TGGCTTACYACAGGYCACATGAAAGT	10^−3^
		MARV FiloNP-Rm GTGTGTGATTTCAGTTTTYTGGAGGTGGAA	FFU/reaction***
	L(Sanchez 1999)	EBOV FiloNP-Fe TGGCAATCAGTDGGACACATGATGGT	
		EBOV FiloNP-Re TGGCAATCAGTDGGACACATGATGGT	
		MARV FILO-A ATCGGAATTTTTCTTTCTCATT	
		MARV FILO-B ATGTGGTGGGTTATAATAATCACTGACATG	
Bergqvist 2015[73] (NB Multiplex)	L	EV F1 Biotin CGTTTIAAIACCMIWCTSATTGC	
		EV F2 Biotin CGATTCAACACAACTCTAATCTC	
		EV F3 Biotin CGATTTAATACTTTACTGATTGC	
		EV F4 Biotin AGGTTIAATACATCACTGATTGC	
		EV R Phosphor GGRTGSCCCCARTGYTTTTGVA	
		EBOV P C_12_-NH_2_ GCATAGACACAATCTTAAAATTG	1500 copies/mL
		SUDV P C_12_-NH_2_ GAGATTGAATATCATCTACCAGT	150 copies/mL
		TAFV P C_12_-NH_2_ GGTCAGACACTGTTTCTGGTA	150k copies/mL
	GP	MV F Biotin ACACYYYCAARHRCAACYTCAGYAC	
		MV R Phosphor TCAAAATCAATYKSAGYAYTTATTAACCCRTC	
		RAVV P C_12_-NH_2_ GCTAGTTCACGTTGTGTATCATT	
		MARV OZOLIN P C_12_—NH_2_ CCAACACACAAAGCATGGCCACTG	
		MARV MUSOKE P GATTGTGCTCTGTGTGTTGTC	
		MARV LEIDEN/POPP P GTGGCTGTGCTCTGTGTGTCGTA	

* where available.

** where antigen detection used as standard (from 26 symptomatic patients, 3 convalescent and 1 healthy).

*** FFU: Focus forming unit.

### 4.2 RT-qPCR

This technique is very similar to RT-PCR but enables the sample to be quantified against a known set of standards. It uses forward and reverse primers and internal oligo probes with fluorophores and quenchers. qPCR machines measure fluorescent signal from probe break down, which occurs relative to amplification, and can detect multiple fluorophores, allowing multiplexing within a single sample (e.g. differentiating between *Ebolavirus* and *Marburgvirus*) [34]. Photon multiplier tubes amplify fluorescence signals, potentially increasing sensitivity over RT-PCR. Furthermore, a large number of samples can also be run at the same time as the standards; depending on the format of the device, 96/384 wells. Examples of these machines include: CFX96 (Biorad), Lightcycler (Roche), ABI 7000 series (Applied Biosystems) and Rotor-Gene Q (Qiagen).

Published RT-qPCR assays for filovirus detection are presented in Table 3. The benchmark that many of the newer tests are compared against is the Trombley assay [35]. The targets described in this paper are the nucleoprotein, glycoprotein and VP40 (matrix protein). Minor groove binding (MGB) probes and standard Taqman™ probes were assessed in this paper for a number of viral pathogens; MGB probes are shorter than normal yet maintain specificity and increased primer melting temperature (relative to similar length Taqman™ probes) by adding a 3′ minor groove binding moiety that stabilises the probe-target hybrid. The Trombley assay includes primers for a human gene (ribonuclease P) as an endogenous control. Due to variation between the five subtypes of Ebolavirus, separate primers and probes are required for each. While this assay has high sensitivity for Zaire Ebolavirus (a lower limit of detection (LLOD) of 0.0001 plaque forming unit per reaction) it is expensive, time consuming and suited predominantly to well-equipped diagnostics laboratories. During the 2014–2016 Ebolavirus a number of RT-qPCR assays were authorised for emergency use (FDA EUA) [36], presented in Table 4. Two platforms used during the epidemic (Biocartis Idylla and Cepheid GeneXpert) have sample-to-result cartridges, whereas the others require sample preparation before the RT-qPCR can be performed. This is of particular interest as using a sample-to-result cartridge can reduce operator involvement, improving safety, exposure to RNases and ease-of-use. The GeneXpert system was used in Liberia during the 2014–2016 outbreak in a mobile laboratory run by Liberian laboratory technicians who had been trained and supported by the Liberian Ministry of Health, WHO and other international partners. Results from the laboratory were used for both clinical management and for determining discharge status of patients [37].

**Table 3:** RT-qPCR papers, target primers/probe and sensitivity for filovirus detection.

Paper	Target gene/sequence	Primers	Sensitivity
Towner (SEBOV)[70]	NP	Reverse transcription (and Forward primer)	One step: 10^3^/ml (1 copy/reaction), Two step: 10^5^/ml (100 copies/reaction)
		F 5′-GA AAGAGCGGCTGGCCAAA-3′.	
		R AACGATCTCCAACCTTGATCTTT	
		P GACCGAAGCCATCACGACTGCAT	
Trombley [35]	EBOV MGB, NP	F565 5′-TCTGACATGGATTACCACAAGATC − 3′	0.001 PFU/reaction
	EBOV, GP	R640 5′-GGATGACTCTTTGCCGAACAATC − 3′ p597S 6FAM-AGGTCTGTCCGTTCAA-MGBNFQ	0.01 (584 copies)
		F2000 5′ −TTTTCAATC CTCAAC CGTAAG GC − 3′	
	SUDV MGB, NP	R2079 5′ − CAGTCC GGT CCCAGAATGTG − 3′	0.1 PFU/reaction
		p2058A 6FAM-CATGTGCCGCCCCATCGCTGC-TAMRA-3′	
	SUDV, GP	F CAT GCA GAA CAA GGG CTC ATT C	0.1 PFU/reaction
		R CTC ATC AAA CGG AAG ATC ACC ATC	
		P CAA CTT CCT GGC AAT	
	RESTV MGB, GP	F AGG ATG GAG CTT TCT TCC TCT ATG	1.0 (34 copies)
		R TAC CCC CTC AGC AAA ATT GAC T	
	RESTV, VP40	P CAG GCT GGC TTC AAC TGT AAT TTA CAG AGG	1 PFU/reaction
		F TCA CCG CGA ACC CAA TG	
		R TCG CTT GTC ATG GTT GGA CTT	
	TEBOV MGB, GP	P ACC ATT GCC C	1.0 (586 copies)
		F CTA TGG TTA TCA CCC AGG ATT GTG	
		R GTA ACT ATC CTG CTT GTC CAT GTG	
	TEBOV, GP	P TGC CAC TCT CCA GCC AGC CAT CCG	0.1 PFU/reaction
		F CCC ATC TCC GCC CAC AA	
		R GAG TGG AAT CCT CTG AAA CCA ATT	
	BDBV, MGB	P CGC AGG CGA AGA C	10^−6^ (RNA dilution)
		F TGT ACA CAA AGT CTC AGG AAC TGG	
		R GTC ATA CAG GAA GAA GGC TCC TTC	
	panMARV MGB, GP	P CCA TGC CCA GGA GGA CTC GCC TTT	0.1 (Ravn), 1.0 (Ci67), 10 (Musoke), 1.0 (Angola) PFU/reaction
	panMARV, GP	F ATG GAA ACC AAG GCG AAA CTG	0.1 (Ravn), 10 (Ci67), 1.0 Musoke), 10 (Angola) PFU/reaction
		R TAC TTG TGG CAT TGG CTT GTC T	
		P CGG GTA GCC CCC AAC	
		F GAT TCC CCT TTG GAA GCA TCT	
		F2 GAT TCC CCT TTA GAG GCA TCC	
		R CAA CGT TCT TGG GAG GAA CAC	
		P ACG ATG GGC TTT CAG	
		F GAT TCC CCT TTG GAA GCA TCT	
		F2 GAT TCC CCT TTA GAG GCA TCC	
		R CAA CGT TCT TGG GAG GAA CAC	
		P AAA CGA TGG GCC TTC AGG GCAGG	
		P2 AAG CGA TGG GCT TTC AGG ACAGG	
Drosten /Sanchez [74,75]	L (MARV and EBOV)	Filo A ATCGGAATTTTTCTTTCTCATT	5.3 copies/reaction (2647 copies/ml (1887 to 4964))
		Filo B ATGTGGTGGGTTATAATAATCACTGACATG	
Gibb [76]	GP	F TGGGCTGAAAAYTGCTACAATC	LOD 8 PFU (10fg*)
		R CTTTGTGMACATASCGGCAC	
		EBOV P CTACCAGCAGCGCCAGACGG	
		SUDV P TTACCCCCACCGCCGGATG	3 PFU (100fg*)
Weidmann [77]		EBOV F ATGATGGAAGCTACGGCG	LOD: 10 copies/reaction Comparable to MARV and EBOV (∼10 copies/reaction)
		EBOV P CCAGAGTTACTCGGAAAACGGCATG	
		EBOV R AGGACCAAGTCATCTGGTGC	
		SUDV F TTGACCCGTATGATGATGAGAGTA	
		SUDV P CCTGACTACGAGGATTCGGCTGAAGG	
		SUDV R CAAATTGAAGAGATCAAGATCTCCT	
		MARV F CAATTCCACCTTCAGAAAACTG	LOD: 10 copies/reaction
		MARV P CACACACAGTCAGACACTAGCCGTCCT	
		MARV R GCTAATTTTTCTCGTTTCTGGCT	

MGB: Minor groove binding. NP: Nucleoprotein. GP: Glycoprotein. L: Polymerase.

* Purified RNA.

**Table 4:** FDA EUA: New technologies. Targets and sensitivity (where published) for EBOV.

Test	Target	Sensitivity	Further notes
Idylla™ Ebola Virus Triage Test[78]	GP, Human RNase P mRNA	465 pfu/mL (1010 copies/mL)	Cartridge, Idylla™ Instrument
Xpert^®^ Ebola Assay[31,[79], [80], [81]]	GP and NP (as well as a sample processing control and human DNA sample adequacy control*)	sensitivity 100%, 95% CI 84.6%–100% vs Trombley	Cartridge, GeneXpert platform
LightMix^®^ Ebola Zaire rRT-PCR Test[82]	L gene (polymerase) and human housekeeping mRNA	4781 PFU/mL	LightCycler^®^ 480 II or cobas z 480 Analyzer
RealStar^®^ Ebolavirus RT-PCR Kit 1.0[34]	L gene and heterologous target sequence	11–67 copies/reaction	Platform dependent sensitivity
(CDC) Ebola Virus VP40 Real-time RT-PCR Assay[51]	VP40	20–60 TCID50/mL (600 TCID50/mL with whole blood)	ABI 7500 Fast Dx Real-Time PCR Instrument, BioRad CFX96
(CDC) Ebola Virus NP Real-time RT-PCR Assay[51]	NP	600–6000 TCID50/mL (6 × 10^3^ TCID50/mL with whole blood)	ABI 7500 Fast Dx Real-Time PCR Instrument, BioRad CFX96
(DoD) EZ1 Real-time RT-PCR Assay[83]	GP, Human RNase P	5000 PFU/mL (7500 PFU/mL with whole blood)	ABI ^®^ 7500 Fast Dx, LightCycler
Liferiver**™** Ebola Virus (EBOV) Real Time RT-PCR Kit[84]	Not stated	23.9/reaction 95% CI (13.4–405.9RNA/reaction)	Roche light cycler 480

* To ensure sufficient host sample has been added.

### 4.3 RT-LAMP

In contrast to standard RT-PCR, reverse transcription-loop mediated isothermal amplification (RT-LAMP) is conducted at one temperature and therefore does not require high precision thermocyclers; this technique is suited to low resource settings. Within a relatively short time period, a very large pool of template can be produced (for in depth methods see [38]). A by-product of the amplification is magnesium pyrophosphate, which can even be seen by eye, and is a useful diagnostic indicator where further analysis, by agarose gel for example, is not available. While purified RNA is generally required for diagnosing filoviral infection, RT-LAMP is influenced to a lesser extent by PCR inhibitors found in the blood and can therefore be used directly with clinical samples [39,40]. As with RT-PCR, one of the main drawbacks of this technique is the inability to multiplex. Published RT-LAMP assays are presented in Table 5.

**Table 5:** RT-LAMP papers, targets, primers and sensitivity for Ebolavirus detection.

Paper	Target	Primers	Sensitivity
Kurosaki [41,42]	Trailer	EBOV F3 CAATAAACAACTATTTAAATAAC	100% (92.5–100) compared to RT-qPCR
		EBOV FIP GTCACACATGCTGCATTGTGTTTTCTATATTTAGCCTCTCTCCCT	
		EBOV BIP AACGCAACATAATAAACTCTGCATTTTATCAATAACAATATGAGCCCAG	
	NP	EBOV B3 CTGGCAAGATATTGATACAACA	97.9% (88.7–100) compared to RT-qPCR
		EBOV LF AATTTTTTGATTATCACGC	
		EBOV F3 TGAAGTCAAGAAGCGTGATGG	
		EBOV FIP CATGGCAGCAAGTGTTCTCTTTTTAGTGAAGCGCCTTGAGGAA	
		EBOV BIP CAGTTTCTCTCCTTTGCAAGTCTTTTTGAACCTTCTCAAGGCAAGCC	
		EBOV B3 AGTCCTTGCTCTGCATGTACT	
		EBOV LF TGTTTTTTCCACTAGATACTGCTGG	
		EBOV LB TCCTTCCGAAATTGGTAGTAGGA	
Xu [85]	GP	EBOV-F3 TGGTTCAAGTGCACAGTCAA	LOD: 30 copies (RNA) ≥10^2^ TCID50/ml (Viral particle)
		EBOV-B3 TGTCTGCTCTACGGTGATGT	
		EBOV-FIP(F1c + F2) GGAGGTTGAGGACTCGTGGAG GGAAGGAAAGCTGCAGTGT	
		EBOV-BIP(B1c + B2) CCAAAACAGGTCCGGACAACAG TCCAACTTGAGTTGCCTCAG	
		EBOV-LF Biotin-GCAAGGGTTGTCAGATGCG	
		EBOV-LB FITC-ATAATACACCCGTGTATAAACTTGAC	
Benzine [44]	GP	EBOV F3 GACGGGAGTGAGTGTCTACC	LOD: 2.8 × 10^2^ PFU/reaction (Kikwit) 1 × 10^3^ PFU/reaction (Makona)
		EBOV B3 AGCTTGGGGCAGTATCAGAA	
		EBOV FL GCACATACCGGCACC	
		EBOV BL CTTCCTGTATGATCGACTTGCTTC	
		EBOV FIP (F1c + F2) GGCACATGGTCCCGTTCCTGATTTTTTAGCGCCAGACGGGATTCG	
		EBOV BIP (B1c + B2) TGCCTTCCACAAAGAGGGTGCTTTTTGCGAAAGTCGTTCCTCGGT	
Oloniniyi [86]	NP	EBOV F CTTAAGAATTCTCACTGATGATGTTGCAGGATTG	256 copies/reaction
		EBOV R CTTAAGGATCCATGGATTCTCGTCCTCAGAAAATC	
		EBOV R1 CTTAAGGATCCATGGATTCTCGTCCTCAGAAAGTC	
		SUDV F CTTAAGAATTCTCAGTCATGTTGAAGAACGGCAAG	
		SUDV R CTTAAGGATCCATGGATAAACGGGTGAGAGGTTC	256 copies/reaction
		BDBV F CTTAAGAATTCTCACCTGTGATGCTGGAGGA	
		BDBV R CTTAAGGATCCATGGATCCTCGTCCAATCAG	
		TAFV F1 ACTATAGGGCGAATTCATGGAGAGTCGGGCCCAC	256 copies/reaction
		TAFV F2 AAGGCTGCCCTTAGCTCGCTAGCACAACATGGAGAG	
		TAFV R2 CGACTCTAGAGGATCCTTACTTGTGGTGCTGAAGG	256 copies/reaction
		RESTV F CTTAAGAATTCTTACTGATGGTGCTGCAAGTTGC	
		RESTV R CTTAAGGATCCATGGATCGTGGGACCAGAAG	64 copies/reaction

RT-LAMP was assessed during the 2014–2016 EBOV outbreak in Guinea for surveillance and was directly compared with RT-qPCR [41]. In this study, buccal swabs (896) from cadavers and a small number of serum samples (21), from individuals with high-risk of EVD (based on contact tracing), were tested with both methods; none of the samples were EBOV positive [41]. The assay had been developed prior to use in Guinea [42,43] and then tested with 100 clinical samples from suspected EVD cases from Guinea. Results were assessed by measuring the turbidity (LA-200 device) of the RT-LAMP reaction or the level of fluorescence in the presence of an inter-chelating dye (Genie III device, Optigene). Compared with RT-qPCR results, the assay was nearly as sensitive (97.9% (95% CI: 88.7–100)) and positive results returned within 25 minutes [42]. Notably these assays used RNA isolated from either buccal swabs or serum. Recently a test was developed that was designed for direct whole blood use [44]. Whole blood is diluted 1:19 in lysis buffer and filtered (10 μm filter) into tubes containing lyophilised RT-LAMP reagents; the authors state that the whole assay takes 40 minutes.

### 4.4 Sequencing

Directly sequencing sample, and comparing to a database, allows direct diagnosis of an infection [45]. As whole or partial fragments of the pathogen are amplified (average read length mid-2015 was 5 kb [46]) and sequenced, this can take longer than RT-qPCR. A major strength of this technique is that, in addition to providing a diagnosis, it allows tracking of pathogen spread, and monitoring for the development of virulence and potential resistance. While sequencing generally requires a well-resourced laboratory with both sequencing machinery and computer analytics, portable systems have been developed. During the 2014–2016 EBOV outbreak a sequencing device, MinION (Oxford Nanopore Technologies) was used in Guinea for sequencing and analysis of 142 EBOV samples [45,46], demonstrating that in-field use of sequencing is feasible. For general surveillance of circulating viruses in a region, next-generating sequencing has been proposed [47,48].

### 4.5 Novel and or secondary diagnostic nucleic acid methods

Novel filovirus nucleic acid-based diagnostics are presented in Table 6. Of these, only one was granted Emergency Use during the 2014–2016 Ebolavirus outbreak was the FilmArray Biothreat E test [49]. This test is similar to RT-qPCR in that it has a reverse transcription step followed by a multiplexed PCR step. However, the products of the initial PCR are distributed to an array of secondary PCRs which use nested (internal) primers in combination with an interchelating fluorescent dye. The final products are measured using a film array. The assay itself is within a self-contained pouch in which the RNA template is released by a combination of chemical and mechanical (bead beating) means prior to RT and PCR. This assay has been tested both in the UK [50] the USA [51] and in field conditions in Sierra Leone [50] and Guinea [52]. Aside from the Biothreat E test, those methods described in Table 6 were not tested during the outbreak but show promise in development of future diagnostics.

**Table 6:** Novel NATs for detection of filoviruses.

Test	Notes
FilmArray Biothreat-E test [[50], [51], [52],^87^]	Whole blood or urine sample. Estimated LoD: 6 × 10^5^ PFU/ml. FDA EUA.
QuRapID platform [53]	In blood RT-qPCR (far red dyes), use in resource poor regions. 20 kg, table top device, car alternator/battery or mains capable.
Virocyt [88]	Flow based particle detection of virus. Fluorescent staining of both genome and protein. EXPERIMENTAL uses; not suited for clinical samples due to high levels of other protein.
Lab-on-chip Optofluidic detection [89]	LoD 0.2PFU/ml. Amplification free by using sample concentration before measurement by laser.
Circulating microRNA [90]	Measuring EBOV induced changes in miRNA in humans and NHP. Proof of principle assay. 36 differentially expressed miRNAs; 93.1% (27/29) accurate in acute cases
Padlock probe detection [91]	Rolling circle amplification (RCA) of EBOV L gene on magnetic beads followed by secondary circle to circle amplification. Combined Detection by biotin capture and magnetic bead for an electrochemical and magnetic actuation. LOD:33 cDNA molecules.
One step FRET-PCR [92]	Multiplex assay differentiating between RT-qPCR products by Tm; 6FAM and LCRed 640 probes. Products from different ebolavirus subtypes had both distinct Tm fluorescence and amplicon size which allows typing.
FILODIAG [62]	Filovirus Diagnostics. Ultra-fast laser amplification using laser-heated, primer coated, nanoparticles for rapid heating/cooling. Aim of 15 min sample-to-result.
Mofina [63]	Portable POC device for the detection of Ebolavirus or Marburg virus. Sample-to-result in 75 min

Utilization of far red fluorophores has been examined with whole blood samples to overcome signal inhibition of blood constituents. The QuRapID system uses these dyes in addition to rapid freeze/thaw cycles to isolate and then amplify viral RNA. The 20 kg stand-alone system has been developed for field use [53]. Two bead-based PCR assays were developed to detect multiple RNA viruses from bat urine [54]. Briefly, a one-step RT-PCR is combined with primers with a 5′ tag (24 nt) and biotinylated dCTP nucleotides. Fluorescently labelled microbeads with an anti-tag sequence then bind to amplification products. A Bio-plex 200 flow cell instrument measures the bead and amplification product. This bead-based technology could be adapted for use in multiplex filovirus diagnostics for humans.

## 5 Point-of-care diagnosis

A significant goal for filovirus diagnostics is the development of point-of care (POC) diagnosis. The ASSURED criteria set out by the WHO for POC devices are:

Affordable, Sensitive, Selective, User-friendly, Rapid, Equipment-free, and Deliverable (to end users) [55]. After the start of the EVD outbreak in 2014, a target product profile for diagnostics for Ebolavirus was proposed [56].

While there are rapid diagnostic tests based on an antibody response to viral antigen(s), an early diagnosis of filoviral infection is preferable, ideally before the humoral response has developed, and nucleic acid testing can do this. Studies in non-human primates have shown that post exposure prophylaxis (PEP) using vaccines for filoviruses can increase rates of survival even 2 days post-exposure [57]. The recent phase III trial of the VSV-ZEBOV vaccine indicates that this may be the case for human infections with EBOV [28]. There is also evidence that PEP with antibodies can be effective in non-human primates [58,59] and murine models [60]; notably a definitive diagnosis of a patient would be required before administration of treatment. Evidence from animal models indicate the earlier the administration of either vaccine or antibodies the greater the survival odds.

Furthermore, in filovirus outbreak situations, POC devices could play a key role in the triage of patients presenting to a clinic with fever. Multiplexed devices could assess whether a patient has multiple infections; for example with a virus as well as malaria and thereby feed into the clinical and therapeutic pathway [61]. A key aspect of POC devices is that there is minimal sample handing and potentially pathogenic material does not require transport to distant sites, thereby improving the diagnostic turnaround time.

Two projects funded via the Innovative Medicine Initiative (IMI) are attempting to address the need for novel near-patient filovirus diagnostics. A device that uses a laser based ultra-fast PCR is being developed by the FILODIAG consortium [62]. This technology utilises primer coated nanoparticles that are rapidly heated by laser absorption and then cool down immediately. This is faster than conventional thermocyclers; the aim is to test for EBOV within 15 min. A POC diagnostic device is being developed by the Mofina consortium for Ebolaviruses and Marburg virus detection. It is small, portable and will deliver results within 75 min following skin prick blood sampling [63]. As such, it will be well suited for in field use during filovirus outbreaks.

## 6 Conclusions

Nucleic acid tests have the greatest potential for early detection of filovirus infection. Their main strength is that only a small amount of input material is required for both detection and typing (either by specific primers/probes or sequencing). These tests can also be used in live vaccine administration to assess viral replication.

While the main focus during an outbreak of filoviral infections is plasma viraemia, other sites of viral persistence have been identified [[64], [65], [66], [67], [68]]. Assessing the ability of the described NATs when starting with a different clinical sample matrix is important. This is even more critical for POC devices where sample is put into a device unprocessed rather than purified RNA. The majority of the tests described in this review have focused on filoviral infections, yet the ideal test would incorporate a number of likely pathogens for a region to allow discrimination between causes of fever. NATs that utilise multiplexing that are integrated with novel POC platforms are eminently suited to this objective and, ultimately, will revolutionise outbreak diagnostics.

## Funding

HMS is supported by the Wellcome Trust Institutional Strategic Support Fund (204809/Z/16/Z) awarded to St. George’s University of London.

## Competing interests statement

JT and ADS work for QuantuMDx, a company developing diagnostic devices. SK is a paid advisor and chairs the infectious diseases advisory board for QuantuMDx. SK and HMS are both shareholders in QuantuMDx. SK and HMS are in receipt of funds from QuantuMDx to develop diagnostic technologies and assays (that have supported DJC).

All authors approved the final manuscript.
